# Effect of a stepped-care intervention delivered by lay health workers on major depressive disorder among primary care patients in Nigeria (STEPCARE): a cluster-randomised controlled trial

**DOI:** 10.1016/S2214-109X(19)30148-2

**Published:** 2019-05-13

**Authors:** Oye Gureje, Bibilola D Oladeji, Alan A Montgomery, Toyin Bello, Lola Kola, Akin Ojagbemi, Dan Chisholm, Ricardo Araya

**Affiliations:** a Department of Psychiatry, College of Medicine, University of Ibadan, Ibadan, Nigeria; b Nottingham Clinical Trials Unit, University of Nottingham, Queen's Medical Centre, Nottingham, UK; c Department of Mental Health and Substance Abuse, World Health Organization, Geneva, Switzerland; d Department of Health Services and Population Research, King's College London, London, UK

## Abstract

**Background:**

Little is known about how to scale up care for depression in settings where non-physician lay workers constitute the bulk of frontline providers. We aimed to compare a stepped-care intervention package for depression with usual care enhanced by use of the WHO Mental Health Gap Action Programme intervention guide (mhGAP-IG).

**Methods:**

We did a cluster-randomised trial in primary care clinics in Ibadan, Nigeria. Eligible clinics were those with adequate staffing to provide various 24-h clinical services and with regular physician supervision. Clinics (clusters), anonymised and stratified by local government area, were randomly allocated (1:1) with a computer-generated random number sequence to one of two groups: an intervention group in which patients received a stepped-care intervention (eight sessions of individual problem-solving therapy, with an extra two to four sessions if needed) plus enhanced usual care, and a control group in which patients received enhanced usual care only. Patients from enrolled clinics could participate if they were aged 18 years or older, not pregnant, and had moderate to severe depression (scoring ≥11 on the nine-item patient health questionnaire [PHQ-9]). The primary outcome was the proportion of patients with remission of depression at 12 months (a score of ≤6 on the PHQ-9, with assessors masked to group allocation) in the intention-to-treat population. This trial is registered with the International Standard Randomised Controlled Trials Number registry (ISRCTN46754188) and is completed.

**Findings:**

35 of 97 clinics approached were eligible and agreed to participate, of which 18 were allocated to the intervention group and 17 to the control group. 1178 patients (631 [54%] in the intervention group and 547 [46%] in the control group) were recruited between Dec 2, 2013, and June 29, 2015, among whom 976 (83%) were female and baseline mean PHQ-9 score was 13·7 (SD 2·6). Of the 562 (89%) patients in the intervention group and 473 (86%) in the control group who completed 12-month follow-up, similar proportions in each group had remission of depression (425 [76%] in the intervention group *vs* 366 [77%] in the control group; adjusted odds ratio 1·0 [95% CI 0·70–1·40]). At 12 months, 17 (3%) deaths, one (<1%) psychotic illness, and one (<1%) case of bipolar disorder in the intervention group, and 16 deaths (3%) and one (<1%) case of bipolar disorder in the control group were recorded. No adverse events were judged to be related to the study procedures.

**Interpretation:**

For patients with moderate to severe depression receiving care from non-physician primary health-care workers in Nigeria, a stepped-care, problem-solving intervention combined with enhanced usual care is similarly effective to enhanced usual care alone. Enhancing usual care with mhGAP-IG might provide simple and affordable approach to scaling up depression care in sub-Saharan Africa.

**Funding:**

UK Medical Research Council.

## Introduction

Depression is a substantial cause of disease burden globally, including in sub-Saharan Africa.^1^, ^2^ Studies done in Nigeria, which has about one psychiatrist for every million people,^3^ have shown that only around one in five people with common mental disorders receive any form of treatment and that, even among those with serious disorders (including those with suicidal behaviour), only around 10% receive minimally adequate treatment.^4^, ^5^ Untreated depression can cause disability, reduce quality of life, increase risk of suicide, increase health-care use and costs, and reduce productivity.^6^

Several studies have shown the effectiveness of a stepped-care approach for treating common mental health disorders in primary care in low-income and middle-income countries (LMICs).^7^, ^8^, ^9^ For example, an efficacy trial showed that a psychological intervention consisting of six to eight behavioural activation sessions delivered by trained lay health workers was, over a 3-month follow-up period, superior to enhanced usual care among patients in primary care with moderately severe to severe depression in India.^10^ In that study, usual care, delivered by physicians, was enhanced with the use of the WHO Mental Health Gap Action Programme intervention guide (mhGAP-IG)—a guide designed to aid non-specialists, especially those working in LMICs, in offering evidence-based treatment for common mental disorders, including depression. Contextualised to the Nigerian health system and pilot-tested,^11^, ^12^ the guide has been adopted by the Nigerian Government for scaling up mental health care in the country's primary care settings.^13^ In another study, among patients in Zimbabwe with common mental disorders (mainly depression and anxiety), a stepped-care, task-shifting approach in which participants received six sessions of problem-solving therapy delivered by lay health workers, with the option of referral to more experienced clinicians for those not improving, was more effective over 6 months than enhanced usual care (enhanced by information, education, and support to the lay health workers).^9^ Problem-solving therapy, a structured but simple psychological intervention that has shown some efficacy in depression trials,^14^, ^15^, ^16^ has been culturally adapted and found to be acceptable to both providers and patients with major depressive disorder in primary care by our group.^17^, ^18^ However, as highlighted in a systematic review, more studies are required to provide empirical evidence for scalable interventions for depression that can be delivered by non-specialist health workers, because studies of adequately robust quality are still few.^19^ More studies are especially needed in settings such as those in much of sub-Saharan Africa where the bulk of primary care services is provided by non-physician, lay health workers.

**Research in context**

**Evidence before this study**

We searched PubMed and PsychINFO from June 1, 2010, to June 30, 2012, for evidence relating to the treatment of depression in primary care in low-income and middle-income countries (LMICs), with no language restrictions. Our search terms included “depression” “treatment/interventions”, “primary care” “non-physician primary care providers”, “low and middle-income countries”, and “lay health workers”. We identified 25 relevant papers, and a further eight by hand-searching the references of those papers. Evidence suggested that primary care providers can, with specialist support, deliver effective interventions for depression that include psychological and medication therapies. However, although the studies showing effectiveness were done in countries classified as LMICs, those countries had much better health-care resources than are obtainable in sub-Saharan countries. For example, studies done in India and Chile were implemented in clinics where routine care was provided by physicians. On the contrary, front-line providers in much of sub-Saharan Africa, including Nigeria, are non-physician lay health workers with much simpler training as health-care providers and with very little experience in the provision of mental health care.

**Added value of this study**

To our knowledge, this is the largest randomised controlled trial of the effectiveness and cost-effectiveness of a stepped-care intervention for moderate to severe major depression consisting of structured problem-solving therapy delivered within enhanced routine primary care services in sub-Saharan Africa. Usual care was enhanced with WHO's Mental Health Gap Action Programme intervention guide (mhGAP-IG) as a clinical support tool. The primary outcome was assessed at 12 months after trial entry, thereby decreasing the likelihood that the outcomes observed were transient. Remission was observed in similar proportions of patients in the group that received enhanced usual care alone and in the intervention group.

**Implications of all the available evidence**

In developing models for integration of mental health care into the busy schedule typical of a primary health service, there is a need to explore the most feasible and affordable pathway to scaling up care for depression in low-resource settings. The mhGAP-IG might offer such a prospect by providing evidence-based assessment and treatment algorithms, including the use of medications, appropriate to non-physician primary care workers who are authorised to prescribe. Future research should address practical issues related to implementing this approach within routine primary care, including how to facilitate detection of depression at this level of care.

The aim of the STEPCARE study was to evaluate the effectiveness and cost effectiveness, over a 12-month period, of a structured problem-solving therapy delivered within a stepped-care approach by non-physician, lay health workers for moderate to severe depression.

## Methods

### Study design and participants

As described in the protocol,^18^ the study was a two-arm, parallel, cluster-randomised, controlled clinical trial conducted in primary care clinics in the city of Ibadan, a large metropolis in the southwest of Nigeria. Among all the primary health-care centres within the city's 11 local government areas (five urban and six rural), those that had a full complement of primary health-care workers (ie, adequate staffing to provide a broad range of 24-h clinical services and with regular physician supervision) were assessed for eligibility. Clinics mainly focused on maternal and child health care in the perinatal period were excluded. The units of randomisation (clusters) were primary care clinics, and the units of analysis were individual participants. Eligible clinics that provided consent to participate were randomly allocated to one of two study groups: a stepped care plus enhanced usual care group (intervention) or an enhanced usual care only group (control).

We recruited consecutive attendees at the enrolled clinics who had a score of 11 or higher on the nine-item patient health questionnaire (PHQ-9),^20^ which has been previously validated in our setting.^18^, ^21^ Other eligibility criteria were ability to speak the study language (Yoruba), age 18 years or older, not being pregnant or breastfeeding, not needing immediate medical attention, and meeting the criteria for a diagnosis of DSM-IV major depression assessed with the short form of the composite international diagnostic interview.^17^, ^22^ Full criteria are described in the protocol.^18^

The study was approved by the University of Ibadan and University College Hospital ethics committee and was monitored by an independent trial steering committee. All participants provided written (or witnessed, if illiterate) informed consent.

### Randomisation and masking

Following recruitment of the initial group of participating clinics, anonymised codes for each clinic were provided by the research team in Ibadan to the study statistician (AAM), who generated the allocation sequence and carried out the random allocation. Primary health-care centres were stratified by local government area and randomly allocated in a 1:1 ratio to the intervention group or the control group. For each local government area, a single balanced block equal to stratum size was generated with use of computer-generated random numbers to ensure balanced allocation to treatment groups. We aimed to recruit 90 participants from each of the 16 clinics initially randomised; however, when recruitment was slower than anticipated, we randomised an additional 19 clinics. Allocation of these additional clinics followed the same procedure.

Outcome assessors were blinded to patients' group allocations, were not involved with screening or recruitment of trial participants in the clinic, and were randomly assigned to participants from any clinic in either group of the study. Data were collected and stored electronically using android tablets and downloaded to a secure server located in the research office. Data were kept anonymously using codes to identify individuals. These datasets did not contain the allocation status of the participants which was kept as a separate file and was available only to the trial statistician.

### Procedures

In the Nigerian setting, front-line primary care providers consist of nurses, community health officers, and community health extension workers, each of whom has 2–3 years of post-secondary school professional training (ie, an average of 14 years' education). Supervision and support to all the primary health-care centres in each local government area (typically eight to ten centres) is provided by a general practitioner, acting as primary health-care coordinator, who runs outpatient clinics, provides clinical supervisions on a scheduled regular basis across the clinics, responds to clinical emergency calls, and has administrative management duties. At each of the participating clinics, two front-line primary care providers, of any cadre (ie, nurse, community health officer, or community health extension worker), were selected and trained to provide treatment appropriate to the study group.

Providers in the intervention group and the control group delivered usual care enhanced with the mhGAP-IG,^23^ in which specifications for the treatment of depression consist of simple psychosocial approaches, including psychoeducation and counselling to address stressors and activate social networks, and pharmacotherapy when necessary. Primary health-care workers in the control group received a 2-day top-up training session in the use of mhGAP-IG. Primary health-care workers in the intervention group were also trained to deliver a structured psychological intervention consisting of behavioural activation (activity scheduling) and problem-solving therapy, previously culturally adapted and pilot tested by us.^17^, ^18^ These providers received 6 days of training on problem-solving therapy and on use of the mhGAP-IG to identify and treat depression, which included didactic lectures, clinical demonstrations, role plays on the delivery of the manualised intervention, procedures for support and supervision by the general practitioner through mobile phones, and how to monitor patients on antidepressant medication. Trained providers had to meet a predefined benchmark for post-training evaluation to be able to participate in the trial. Of the 39 potential providers trained, three did not achieve the set of competency standards and were excluded from the study. A few months into the trial, two trained research supervisors (coordinated by BDO) did a fidelity assessment through direct observation and rating of a total of 205 randomly selected sessions (around six sessions per health worker), using a checklist that consisted of key items of the intervention procedures. Items were rated as 0 (poor or not done), 1 (fair or partially done), or 2 (good or well done).

Consecutive patients at participating primary health-care centres were screened on the PHQ-9 by trained research assistants. Each consenting participant who screened positive (scored ≥11 on the PHQ-9) was provided with their PHQ-9 score and referred to one of the workers providing trial treatment in the clinic.

In the intervention group, in the first step, primary health-care workers use participants' PHQ-9 screen scores to determine treatment options: those with scores of 11–14 were offered eight sessions of psychological intervention delivered by primary health-care workers, and those with scores of 15 or more at baseline were assessed for additional antidepressant medication in consultation with the supervising general practitioner. Therapy sessions were done in person and individually. During the first session, participants were offered psychoeducation in which information about the symptoms of depression, possible causes, and treatments were discussed. After providing reassurance about the treatability of their condition, the structure of the subsequent sessions was discussed and negotiated. The following five sessions dealt with identified problems, difficulties, and stressors, with the therapist working with the patient to explore potential feasible solutions, including how to harness the assistance of supportive social networks. In the final two sessions, both therapist and patient worked together to integrate the experiences of the previous sessions, draw concrete lessons, and use these to prepare for the future. After the first eight sessions, each participant was reassessed with the PHQ-9, this time by the primary health-care worker. Those with a PHQ-9 score of 11 or more, or greater than 50% of baseline score, proceeded to step two. Step two consisted of additional therapy sessions or a combination of therapy and medication following a review by the supervising physician. All participants who did not improve after step two had their cases discussed with a psychiatrist in the third (final) step.

In the intervention group, all supervision and consultations with doctors were provided on as-needed basis and through mobile phone contact, except when a face-to-face review was deemed necessary and feasible. The components and tasks for each treatment session and the clinical decisions and steps were detailed in manuals and charts provided to the primary health-care worker and the primary care physicians. When medication was required, the first-line antidepressant was amitriptyline, which non-physician primary care providers in Nigeria are authorised to prescribe. The trial manual stipulated that, when antidepressant medications were prescribed, the frontline clinician consults with the general practitioner either face-to-face or via mobile telephone to receive appropriate advice on dosing and monitoring. Other antidepressants could be prescribed by the general practitioners for patients who did not improve or had other contraindications to the use of tricyclic antidepressants. Any emergent medication side-effects were reviewed in consultation with the general practitioner.

Participants in the control group received enhanced usual care alone. The choice of intervention (either unstructured psychological treatment or medications as stipulated in the mhGAP-IG) was at the discretion of the primary health-care worker and no specification as to the number of sessions was made.

Outcome assessments were done through face-to-face interviews at the respondents' homes. All outcome assessors were college-educated, experienced in the conduct of surveys, and received 2 weeks of training, including trial life interviews, inter-rater exercises, and regular field debriefing. Interviewers assessed patients' disability level using the WHO disability assessment schedule (WHODAS) 2.0,^24^ and quality of life using the WHO quality-of-life questionnaire (WHOQOL).^25^ The service utilisation questionnaire^26^ was used to collect resource-use data, including any inpatient care, consultations with health providers, use of drugs and laboratory tests, and time and travel costs associated with this service uptake. We obtained information on the financing sources for each of the categories to allow for an estimation of the extent of private, out-of-pocket expenditures incurred by study participants and their families. The unit costs or prices of these various resource inputs were derived through a costing analysis in some participating health facilities with use of data collection templates and protocols previously developed and applied by us.^27^ Yoruba versions of all the questionnaires were derived by standard protocols of iterative back-translations, as done in previous surveys, achieving good psychometric results.^24^, ^28^

### Costs and cost effectiveness

To assess cost effectiveness at follow-up, changes in service costs were analysed with respect to changes on the PHQ-9 and WHODAS at the 6-month and 12-month follow-up visits. The service costs incurred in each group were collected with the service utilisation questionnaire. A set of unit costs and prices for all inpatient and outpatient service use, as well as the costs of the interventions, were generated using simplified costing templates and local data inputs. Cost-effectiveness acceptability curves were constructed for a unit improvement on PHQ-9 and WHODAS.

### Outcomes

The primary outcome was the proportion of patients who had remission of depression (predefined as PHQ-9 score of <6) at 12 months from entry into the trial. Secondary outcomes included depression symptoms as a continuous PHQ-9 score, assessed at 3 months, 6 months, 9 months, and 12 months, as well as level of disability (assessed with the WHODAS),^24^ quality of life (assessed with the WHOQOL),^25^ and health-care use (assessed with the service utilisation questionnaire),^26^ at 6 months and 12 months. All outcomes were assessed in all enrolled patients.

### Statistical analysis

Informed by the results of our pilot study,^17^ we sought to detect an absolute difference of 18 percentage points (59% in the intervention group and 41% in the control group; equivalent odds ratio [OR] 2·1) at 12 months. We assumed an intracluster correlation coefficient of 0·05, also based on pilot study data, and non-collection of the primary outcome for 20% of participants. The uninflated sample size required 131 participants per group for analysis to detect a difference of 59% versus 41% with 80% power and a two-sided α of 5%. We originally aimed to recruit 90 individuals per clinic. With 72 participants per cluster for analysis and an intracluster correlation coefficient of 0·05, the design effect was 4·55, giving a total number required for analysis of 1190. We therefore aimed to recruit 1440 individuals from 16 clinics. However, because participant recruitment was slower than anticipated, we recruited and randomised a further 12 clinics in March, 2014, and seven in November, 2014, giving a total of 35 clinics randomised in the study. Although the design effect was reduced with an increased number of smaller clinics, the total target sample remained 1440.

The primary approach for comparative analyses was to analyse on an intention-to-treat basis at the individual level without imputation of missing data. We used descriptive statistics to assess balance between the study groups at baseline for both clinic and individual participant characteristics. To take appropriate account of the hierarchical nature of the data, we used multivariable mixed-effects regression models to estimate remission from depression at 12 months for the intervention group versus the control group. We present unadjusted and adjusted estimates. In both analyses, we accounted for clustering with use of a mixed-effects model with clinic and local government area as random effects. In the adjusted estimates, we also adjusted for baseline PHQ-9 score, age, and primary health-care centre, and for local government area as a stratification variable. In a sensitivity analysis, we imputed missing primary outcome data with multiple imputation. Similar analyses were repeated for secondary outcomes. For these secondary continuous outcomes, we estimated the difference in mean scores between the intervention and control groups. We also investigated whether between-group differences varied over time by using data from all follow-up visits in repeated-measures analyses. We investigated whether there was any differential effect of the intervention on the primary outcome according to baseline symptom severity (PHQ-9 score <16 *vs* ≥16) by including appropriate interaction terms in the primary regression model. Because the trial was powered to detect overall differences between groups rather than interactions of this kind, these results are to be interpreted with caution. For the cost and cost-effectiveness analyses, 95% CIs were derived through non-parametric bootstrapping techniques owing to the non-normal distribution of mean service costs per study participant (1000 resamples were run). All analyses were done in STATA (version 13.0) software.

This study is registered with the International Standard Randomised Controlled Trials Number registry, number ISRCTN46754188.

### Role of the funding source

The funder of the study had no role in the study design, data collection, data analysis, data interpretation, or writing of the report. The corresponding author had full access to all the data in the study and had final responsibility for the decision to submit for publication.

## Results

Between Sept 2 and Sept 20, 2013, we approached 97 of the 186 primary health-care clinics in Ibadan. 52 of these clinics were ineligible (maternal and child health care clinics) and ten declined to participate. The remaining 35 were randomly allocated: 18 to the intervention group and 17 to the control group (figure). Recruitment of trial participants started on Dec 2, 2013, and ended on June 29, 2015. The last follow-up assessment was completed on July 15, 2016. 8005 consecutive patients were screened in the intervention group, of whom 717 (9·0%) screened positive on the PHQ-9, and 631 (7·9%) were enrolled into the trial. In the control group, 624 (8·6%) of 7278 screened patients scored 11 or higher on the PHQ-9, and 547 (7·5%) were enrolled into the trial (figure). Follow-up at 12 months was completed for 562 (89%) of 631 in the intervention group and 473 (86%) of 547 in the control group. Few patients declined participation. The most common reasons for attrition in both groups were relocation, non-availability after multiple efforts (at least four attempts) were made to conduct outcome interviews, and death (figure). No demographic or clinical features were associated with refusal to participate or with attrition.

**Figure:**
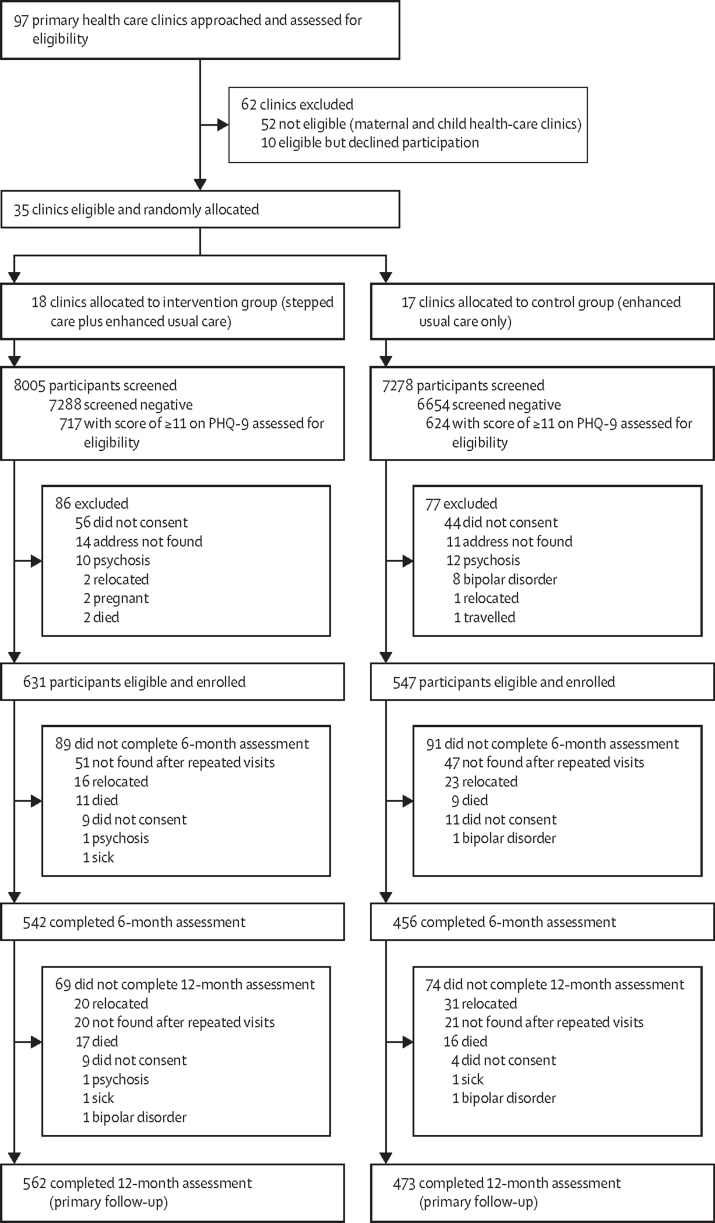
Trial profile

Participant characteristics were well balanced between the two groups at baseline except for mean age, which was higher in the intervention group (table 1). 976 (83%) of 1178 participants were women. Most participants were either unemployed or had unskilled jobs. The mean score of 13·7 (SD 2·6) on the PHQ-9 at baseline suggested that, across the two groups, most participants had at least a moderate severity of depression.

**Table 1:** Baseline demographic and clinical characteristics

		**Intervention group (n=631)**	**Control group (n=547)**
Age, years		50·2 (15·0)	44·0 (14·5)
Years of education		6·8 (5·4)	7·4 (5·5)
Sex			
	Male	104 (16%)	98 (18%)
	Female	527 (84%)	449 (82%)
Marital status			
	Single	95 (15%)	99 (18%)
	Married	486 (77%)	410 (75%)
	Separated or divorced	50 (8%)	38 (7%)
Employment status*			
	Unemployed	66 (10%)	49 (9%)
	Housewife	7 (1%)	11 (2%)
	Unskilled labourer	387 (61%)	341 (62%)
	Skilled labourer	105 (17%)	91 (17%)
	Middle-level worker	51 (8%)	30 (5%)
	Professional	6 (1%)	9 (2%)
	Unknown	9 (1%)	16 (3%)
PHQ-9 score†		13·7 (2·6)	13·5 (2·6)
WHODAS score‡		25·4 (9·2)	25·8 (9·6)
WHOQOL scores§			
	Physical domain	46·6 (21·4)	48·0 (21·9)
	Psychological domain	53·6 (20·4)	51·6 (19·6)
	Social domain	50·8 (25·6)	49·2 (25·3)
	Environmental domain	50·7 (16·4)	47·3 (16·7)

Data are mean (SD) or n (%). PHQ-9=nine-item patient health questionnaire. WHODAS=WHO disability assessment schedule. WHOQOL=WHO quality-of-life questionnaire.

* Employment statuses were defined as follows: unemployed=not currently in paid employment; housewife=woman who is a homemaker and not seeking employment outside the home; unskilled labourer=worker who has not learnt any trade; skilled labourer=artisan; middle-level worker=clerical or secretarial staff, junior admin worker, etc; professional=teacher, nurse, doctor, senior admin staff, etc.

† PHQ-9 scores range from 0 to 27 (nine questions, each scored from 0 [best] to 3 [worst]).

‡ WHODAS scores range from 12 to 60 (12 questions each ranging from 1 [best] to 5 [worst], plus three additional questions about number of days in the past month the patients has had difficulties).

§ WHOQOL includes 26 questions (with responses ranging from not at all or very dissatisfied or very poor, to very much or very good or very satisfied or all the time); scores for each domain are transformed to a scale of 0–100, with higher scores denoting better quality of life.

Each session of problem-solving therapy lasted 30–40 min, with the first session taking slightly longer on average (mean 45 min [SD 10]). 183 (89%) of 205 sessions were rated as substantially adequate in the fidelity assessments.

Of the 631 patients who consented to the trial in the intervention group, 602 (95%) received at least one therapy session, 499 (83%) completed four sessions, and 476 (79%) completed the eight prescribed sessions in step one. Two additional therapy sessions in step two were received by 122 (19%) participants. 76 (12%) participants in the intervention group were prescribed antidepressant medications (amitriptyline): 70 (11%) commenced medication because of severe depression PHQ-9 score >15) at trial entry, and six (1%) started medication following poor response after eight therapy sessions, as specified in the protocol. In the control group, 144 (32%) of 448 participants for whom complete treatment details were available were prescribed amitriptyline. Receipt of counselling from other carers (mainly traditional or faith healers) was reported by 27 (4%) participants in the intervention group and 31 (7%) of 448 in the control group. Only two patients were referred to psychiatrists, both in the control group.

The groups showed no difference in terms of the primary outcome (remission at 12 months: 76% in the intervention group *vs* 77% in the control group; adjusted OR 1·0 [95% CI 0·7–1·4]), and this did not change when multiple imputation was used to account for missing outcome data (table 2). Mean PHQ-9 scores were 4·7 (SD 4·5) at 3 months, 3·8 (4·1) at 6 months, 3·6 (4·2) at 9 months, and 3·6 (4·2) at 12 months for the intervention group, and 4·8 (4·2) at 3 months, 4·3 (4·5) at 6 months, 3·9 (4·4) at 9 months, and 3·5 (3·9) at 12 months for the control group. The adjusted mean difference in PHQ-9 score (adjusted for baseline PHQ-9 score and local government area) in the intervention group compared with the control group across all follow-up timepoints was −0·3 (95% CI −0·7 to 0·1, p=0·163).

**Table 2:** Primary analysis and sensitivity analyses

	Remission*at 12 months		**Unadjusted analysis**		**Adjusted analysis**†	
	Intervention group	Control group	OR	p value	OR	p value
Non-imputed outcomes	425/562 (76%)	366/473 (77%)	0·9 (0·7–1·2)	0·507	1·0 (0·7–1·4)	0·948
Multiple imputation of missing outcomes	481/631 (76%)	420/547 (77%)	..	..	0·95 (0·71–1·29)	0·760

Data are n/N (%) or OR (95% CI). Intracluster correlation coefficient for primary outcome 0·04. OR=odds ratio. PHQ-9=nine-item patient health questionnaire.

* Defined as PHQ-9 score less than 6.

† Adjusted for baseline PHQ-9 score, age, primary health-care centre, and local government area that participants belonged to; primary health-care centre and local government area were included as random effects.

Secondary outcomes at 6 months and 12 months are shown in table 3. In the adjusted analysis, depression symptoms were less severe in terms of PHQ-9 score in the intervention group than in the control group at 6 months. Mean psychological quality of life score at 6 months was slightly higher in the intervention group than in the control group (unadjusted difference 2·9 [95% CI −0·1 to 5·8]; p=0·058). No differences were seen in any other secondary outcome at either timepoint.

**Table 3:** Secondary outcomes at 6 months and 12 months

			**Intervention group**	**Control group**	**Unadjusted analysis**		Adjusted analysis*	
					Mean difference	p value	Mean difference	p value
6 months			(n=542)	(n=456)				
	PHQ-9 score†		3·8 (4·1)	4·3 (4·5)	−0·5 (−1·1 to 0·0)	0·056	−0·7 (−1·3 to −0·2)	0·011
	WHODAS score‡		18·3 (8·8)	19·5 (9·8)	−0·9 (−2·7 to 0·9)	0·319	−0·9 (−2·7 to 0·9)	0·314
WHOQOL scores§								
		Physical domain	67·8 (20·9)	67·2 (22·2)	0·5 (−3·4 to 4·4)	0·794	0·6 (−3·2 to 4·5)	0·747
		Psychological domain	61·2 (20·1)	58·1 (21·1)	2·9 (−0·1 to 5·8)	0·058	2·9 (−0.1 to 6.0)	0·061
		Social domain	60·3 (19·9)	58·6 (20·7)	2·3 (−0·7 to 5·4)	0·136	2·3 (−0·8 to 5·3)	0·142
		Environmental domain§	57·6 (15·3)	55·0 (16·1)	1·8 (−1·1 to 4·8)	0·224	1·9 (−1·1 to 4·8)	0·214
12 months			(n=562)	(n=473)				
	PHQ-9 score†		3·6 (4·9)	3·5 (3·9)	0·1 (−0·4 to 0·6)	0·808	−0·2 (−0·7 to 0·3)	0·398
	WHODAS score‡		17·9 (8·9)	18·5 (9·8)	−0·3 (−2·2 to 1·6)	0·729	−0·4 (−2·3 to 1·5)	0·708
	WHOQOL scores§							
		Physical domain	72·7 (20·0)	72·5 (20·3)	0·1 (−2·5 to 2·7)	0·950	0·1 (−2·6 to 2·8)	0·918
		Psychological domain	63·9 (20·1)	61·7 (19.6)	2·1 (−0·9 to 5·2)	0·170	2·1 (−0·9 to 5·2)	0·171
		Social domain	65·2 (15·9)	64·7 (17·5)	0·5 (−2·0 to 3·0)	0·692	0·5 (−2·0 to 3·0)	0·696
		Environmental domain	61·1 (15·4)	59·4 (15·1)	1·5 (−0·8 to 3·8)	0·204	1·5 (−0·8 to 3·8)	0·204

Data are mean (SD) or mean difference (95% CI). PHQ-9=nine-item patient health questionnaire. WHODAS=WHO disability assessment schedule. WHOQOL=WHO quality-of-life questionnaire.

* Adjusted for baseline PHQ-9 score, primary health-care centre, and local government area that participants belonged to; primary health-care centre and local government area were included as random effects.

† PHQ-9 scores range from 0 to 27 (nine questions, each scored from 0 [best] to 3 [worst]).

‡ WHODAS scores range from 12 to 60 (12 questions each ranging from 1 [best] to 5 [worst], plus three additional questions about number of days in the past month the patients has had difficulties).

§ WHOQOL includes 26 questions (with responses ranging from not at all or very dissatisfied or very poor, to very much or very good or very satisfied or all the time); scores for each domain are transformed to a scale of 0–100, with higher scores denoting better quality of life.

At 12 months, 17 deaths had occurred in the intervention group and 16 in the control group. Information obtained from patients' close relatives and medical records, when available, suggested that the causes of death were complications of hypertension or heart disease (eight [24%] of 33 patients), tuberculosis (six [18%]), diabetes (two [6%]), asthma (two [6%]), liver failure possibly related to alcoholism (three [9%]), typhoid fever (two [6%]), and cancer (one [3%]). The causes were ascribed to old age or were unknown in nine (27%) patients. Only two (6%) of the deceased patients were on antidepressant medication at the time of death. At the 12-month assessment, one (<1%) patient had developed a psychotic illness and one (<1%) other had developed bipolar disorder in the intervention group, and one (<1%) had developed a bipolar disorder in the control group. Suicidal ideation was reported by 57 (10%) participants in the intervention group and by 66 (14%) of those in the control group. No adverse events were judged by the independent trial steering committee to be related to the study procedures.

Estimated service costs per participant per month fell from 11 392 naira at baseline to 3002 naira (at 6 months) and 1729 naira (at 12 months) in the intervention group, and from 7254 naira to 1981 naira (at 6 months) and further to 1664 naira (at 12 months) in the control group (table 4). Over the full 12-month period from baseline, the estimated costs per patient were 28 129 naira in the stepped-care intervention group and 27 514 naira in the control group. Using the 2016 conversion rate of 150 naira to US$1, these costs translate to $187·50 in the intervention group (mean $15·63 per month) and $183·40 ($15·28 per month) in the control group.

**Table 4:** Costs and cost effectiveness of stepped care and enhanced usual care interventions at 6-month and 12-month visits

		**Baseline**	**6 months**	**12 months**
**Cost analysis**				
Service cost, naira per participant per month				
	Intervention group, mean (SD)	11 392 (68 288)	3002 (14 770)	1729 (5864)
	Control group, mean (SD)	7254 (28 714)	1981 (7166)	1664 (6616)
	Mean difference (95% CI)*	..	1021 (−211 to 2514)	65 (−766 to 793)
Change in cost since baseline				
	Intervention group, mean (SE)	..	−8390 (2767)	−9663 (2711)
	Control group, mean (SE)	..	−5273 (1351)	−5590 (1176)
	Mean difference (SE)	..	−3117 (3156)	−4073 (2923)
**Cost-effectiveness analysis**				
Incremental cost-effectiveness ratio (95% CI) based on PHQ-9		..	4454 (−3235 to 13 419)	40 727 (−12 828 to 102 245)
Incremental cost-effectiveness ratio (95% CI) based on WHODAS		..	3896 (−3508 to 12 704)	20364 (−6302 to 50 330)

PHQ-9=nine-item patient health questionnaire. WHODAS=WHO disability assessment schedule.

* Intervention group versus control group, adjusted for baseline costs.

Over the follow-up period, the stepped-care intervention group showed marginally better symptomatic and functional status improvements than those of the control group, but considerably greater reductions in service costs (table 4). The reduction in cost per one-point improvement on the PHQ-9 with the stepped-care intervention compared with the control was 4454 naira (95% CI −3235 to 13 419) or about $30 at 6 months, and 40 727 naira (−12 828 to 102 245) or about $272 at 12 months. With respect to disability, the reduction in cost per one-point improvement on the WHODAS in the stepped-care intervention group compared with the control group was 3896 naira (−3508 to 12 074) or about $26 at 6 months, and 20 364 naira (−6302 to 50 330) or about $136 at 12 months (table 4). Although the point estimates suggested that the stepped-care intervention plus enhanced usual care might be a more cost-effective intervention than enhanced usual care alone, there was a high degree of uncertainty around these estimates. We also produced a descriptive cost table, scatterplots, and acceptability curves, which revealed a probability of 55–60% for the intervention being a cost-effective approach compared with usual care once a willingness-to-pay level of 50 000 naira ($333) was reached (appendix).

## Discussion

In this study of patients in primary care with major depressive disorder of at least moderate severity, the proportion of patients with remission of depression at 12 months was similar among patients who received a structured, stepped-care intervention consisting of culturally adapted problem-solving therapy and among patients who received usual care enhanced with the use of mhGAP-IG. Participants in the stepped-care intervention group had lower mean PHQ-9 scores at 6 months than those in the control group, a difference that is unlikely to be clinically important. The stepped-care intervention combined with enhanced usual care was found to lower costs more than enhanced usual care alone, with some evidence for a more favourable cost-effectiveness profile for treating depression in this setting. Both groups were similar with respect to functional status (as measured with the WHODAS 2·0) and health-related quality of life (assessed with the WHOQOL-BREF) at the 6-month and 12-month follow-up visits.

The essential component of the treatment in the intervention group was a culturally adapted form of problem-solving therapy which, in a pilot study, had been shown to be acceptable to both providers and patients. In the current study, most participants in the intervention group received eight structured sessions of the therapy in the first step and a few went on, depending on symptom relief, to receive further therapy sessions in the second step or were prescribed antidepressants in consultation with a physician. On the other hand, participants in the control group received a few sessions of basic psychosocial treatment, while many more were prescribed antidepressants than were those in the intervention group. The two groups of participants had essentially the same outcomes.

Few studies in sub-Saharan Africa are available to which the results of our study could be compared, the most comparable being one conducted in Zimbabwe.^9^ Although the follow-up was 6 months in that trial and the participants had much milder depression than those in the current study (61·8% scored 11 or more on the PHQ-9 at baseline, compared with 100% in our study), both studies compared an intervention consisting of several sessions of problem-solving therapy with enhanced usual care. The 6-month outcomes in the intervention groups of both studies were broadly similar (mean PHQ-9 scores 4·5 in the Zimbabwe study and 4·3 in our study), whereas the outcome of the control group in the current study was much better than that of the Zimbabwe study (mean PHQ-9 scores 3·8 and 11·0). The enhancement of usual care with mhGAP-IG and the associated frequent use of antidepressants by the providers in the control group of the current trial might be the reason for the differential outcome. A 2014 systematic review highlighted the importance of assessment and treatment guidelines as enablers of successful task-shifting.^29^ Our observation of high and relatively early remission rates is similar to the 64% remission rate at 3 months in the intervention group of the previous trial in India,^10^ and is consistent with the evidence provided by Ilardi and Craighead^30^ that as much as 60–70% of the total improvement achieved through cognitive-behavioural treatment for depression occurs in the first 4 weeks of therapy.

To our knowledge, our study is the largest in an LMIC setting to evaluate the effectiveness and cost effectiveness of interventions for depression delivered by non-physician primary care workers in which follow-up was over a 12-month period, thus increasing the likelihood that the observed outcomes were not transient. Trial participants had a rigorously defined diagnosis of major depression of at least moderate severity, with scores on a validated screening tool complemented with a structured diagnostic interview. The follow-up rate was high and, with a narrow 95% CI in the primary analysis (0·7–1·4) that excluded the effect the trial was designed to detect (OR 2·1), there is assurance of adequate precision. We used a form of problem-solving therapy that had been previously culturally adapted and piloted in our setting. Treatments were delivered by currently serving primary care workers and no new cadres of staff were specially recruited. This has the advantage of enhancing appropriateness to routine primary care in LMICs, thus making sustainability and policy adoption of the findings more likely. Outcomes were assessed by assessors masked to group allocation, with validated tools that had been subjected to cultural adaptation. However, given the complex nature of the treatments in both groups, it is difficult to be sure about the specific aspects of the interventions that were responsible for the change in the depression status of the patients. This is a common observation in studies exploring complex interventions in primary care.^9^, ^10^, ^15^ For the intervention group, although problem-solving therapy has been shown to be an effective treatment for depression, the frequency of contacts with providers might have been important in bringing about remission. In the control group, as previously highlighted in a systematic review,^29^ the enhancement of usual care by an evidence-based assessment and treatment algorithm that facilitated the use of antidepressants by the providers must have been of importance, leading to high remission rates. Given the similarity in the outcomes in both groups of this study, we can infer that the more intensive psychological treatment in the intervention group was probably counterbalanced by a greater use of antidepressant medication in the control group.

The results of this trial should be interpreted with due consideration for the limitations. First, about one in five eligible clinics declined participation in the trial. Second, the intervention was compared with enhanced usual care rather than no treatment. This comparison is similar to the approach taken in many other studies.^9^, ^15^ In this study, usual care was enhanced with use of an evidence-based clinical support tool recently adopted as a pathway to scaling up mental health care in the country. The participants in the control group thus received more intensive care than they would have received before that new policy. The observation that as many as 32% of patients in the control group received antidepressant medication attests to this enhancement. Typically, patients with depression would not be prescribed antidepressant medications and would receive, when identified, non-specific counselling, vitamins, or sleep tablets.^31^, ^32^ A third limitation was our inability to detect a difference between the two study groups, which might reflect methodological issues. For example, although a great majority of the providers were rated to have performed well, there was still variability in the level of performance. Fourth, the much higher proportion of women in the sample, although reflecting in part the profile of clinic population and the higher occurrence of depression in women, might limit the generalisation of findings to men. Fifth, we did the fidelity assessment on some but not all therapy sessions in the intervention group and none in the control group; therefore, we cannot be sure of the adequacy of the problem-solving therapy delivery in the non-assessed sessions in the intervention group or whether providers in the control group also provided sessions that had features of problem-solving therapy. The adequacy of the assessed sessions in the intervention group might also have been partly due to the possibility that providers took extra care because of the presence of the assessor.

In summary, for patients presenting with moderate to severe depression in primary care settings in sub-Saharan Africa, a structured problem-solving therapy delivered within a stepped-care approach combined with usual care (enhanced with use of the WHO mhGAP-IG) might provide similar benefit in terms of remission at 12 months, and might represent a more cost-effective intervention compared with enhanced usual care alone. Further research is warranted to confirm these findings and to elucidate whether, using the mhGAP-IG, non-physician health workers in routine primary health-care settings in LMICs could detect and assess depression and offer appropriate treatment.

## Data sharing

The deidentified participant data on which this report is based will be made available, following publication, upon request to ogureje@comui.edu.ng, and after a signed data access agreement with the principal investigator.
