# Supplementary material for: Effect of a stepped-care intervention delivered by lay health workers on major depressive disorder among primary care patients in Nigeria (STEPCARE): a cluster-randomised controlled trial
Source: Lancet Glob Health. 2019 May 13;7(7):e951–60. doi: 10.1016/S2214-109X(19)30148-2 (PMC6559947; doi:10.1016/S2214-109X(19)30148-2)

THE LANCET
Global Health

Supplementary appendix

This appendix formed part of the original submission and has been peer reviewed. We post it as supplied by the authors.

Supplement to: Gureje O, Oladeji BD, Montgomery AA, et al. Effect of a stepped-care intervention delivered by lay health workers on major depressive disorder among primary care patients in Nigeria (STEPCARE): a cluster-randomised controlled trial. *Lancet Glob Health* 2019; published online May 13. http://dx.doi.org/10.1016/S2214-109X(19)30148-2.

Web Table 1: Mean (SD) cost incurred by each provider across the different assessments by treatment arms.

	Baseline		3 months		6 months		9 months		12 months	
	**STEP**	**EUC**	**STEP**	**EUC**	**STEP**	**EUC**	**STEP**	**EUC**	**STEP**	**EUC**
**In-patient services**<br>Hospital admission	**491 (3677)**	**467 (3336)**	**577 (4379)**	**658 (8010)**	**1012 (9043)**	**542 (4653)**	**342 (4159)**	**1172 (12202)**	**470 (3042)**	**354 (2428)**
**Out-patient services**<br>Nurse	689 (912)	820 (994)	311 (922)	152 (671)	222 (689)	170 (560)	107 (430)	53 (240)	199 (665)	173 (764)
Doctor	3048 (47130)	987 (12791)	-	-	-	-	-	-	-	-
PHC provider	193 (554)	253 (624)	983 (1706)	283 (674)	463 (842)	241 (619)	360 (663)	272 (539)	282 (645)	253 (718)
Psychiatrist doctor	180 (4526)	243 (4927)	-	78 (1723)	-	145 (2216)	-	-	101 (2398)	-
OMH/social worker	11 (147)	2 (30)	36 (292)	74 (309)	49 (321)	103 (413)	149 (494)	125 (398)	50 (205)	42 (197)
Day care/group support	6 (139)	2 (38)	11 (144)	15 (200)	2 (29)	27 (236)	2 (38)	4 (82)	-	-
Hospital emergency	3 (71)	116 (1913)	4 (56)	26 (305)	19 (258)	8 (97)	21 (283)	25 (306)	15 (296)	17 (192)
**Total outpatient costs**	**4131 (47347)**	**2423 (13939)**	**1345 (1796)**	**628 (2013)**	**755 (1043)**	**693 (2440)**	**638 (984)**	**479 (823)**	**647 (2552)**	**486 (1074)**
**Transport cost**	**107 (139)**	**95 (237)**	**108 (319)**	**47 (98)**	**71 (123)**	**43 (119)**	**61 (122)**	**43 (108)**	**54 (114)**	**46 (128)**
**Drug cost**	**691 (1477)**	**658 (1472)**	**692 (2337)**	**513 (2531)**	**454 (1487)**	**546 (1967)**	**430 (1615)**	**609 (2621)**	**456 (2426)**	**523 (2317)**
**Time cost**	**5972 (46094)**	**3612 (23097)**	**164 (2591)**	**565 (6756)**	**711 (9800)**	**157 (2965)**	**289 (3837)**	**813 (8065)**	**102 (1482)**	**255 (4980)**
**TOTAL COST**	**11392 (68288)**	**7254 (28714)**	**2885 (6718)**	**2410 (13034)**	**3002 (14770)**	**1981 (7166)**	**1760 (7421)**	**3117 (19787)**	**1729 (5864)**	**1664 (6616)**

Web Figure 1: Scatterplot of incremental costs (Naira) and effects (PHQ-9) between STEP versus EUC at 12 months

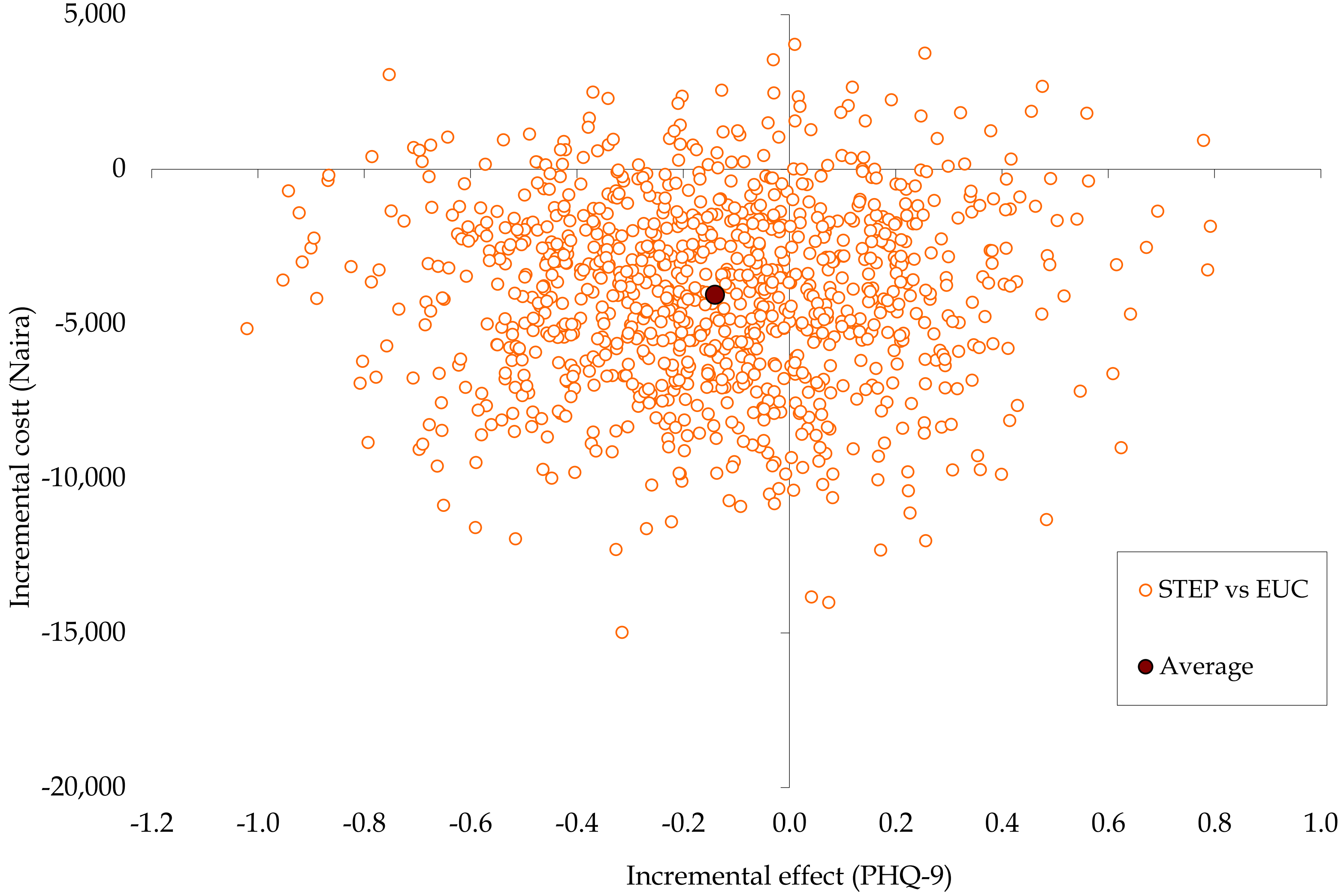

Web Figure 2: Cost-effectiveness acceptability curve showing probability that STEP is a cost-effective intervention compared to EUC at different levels of willingness-to-pay for a one point improvement on PHQ-9 (in Naira) at 12 months follow-up

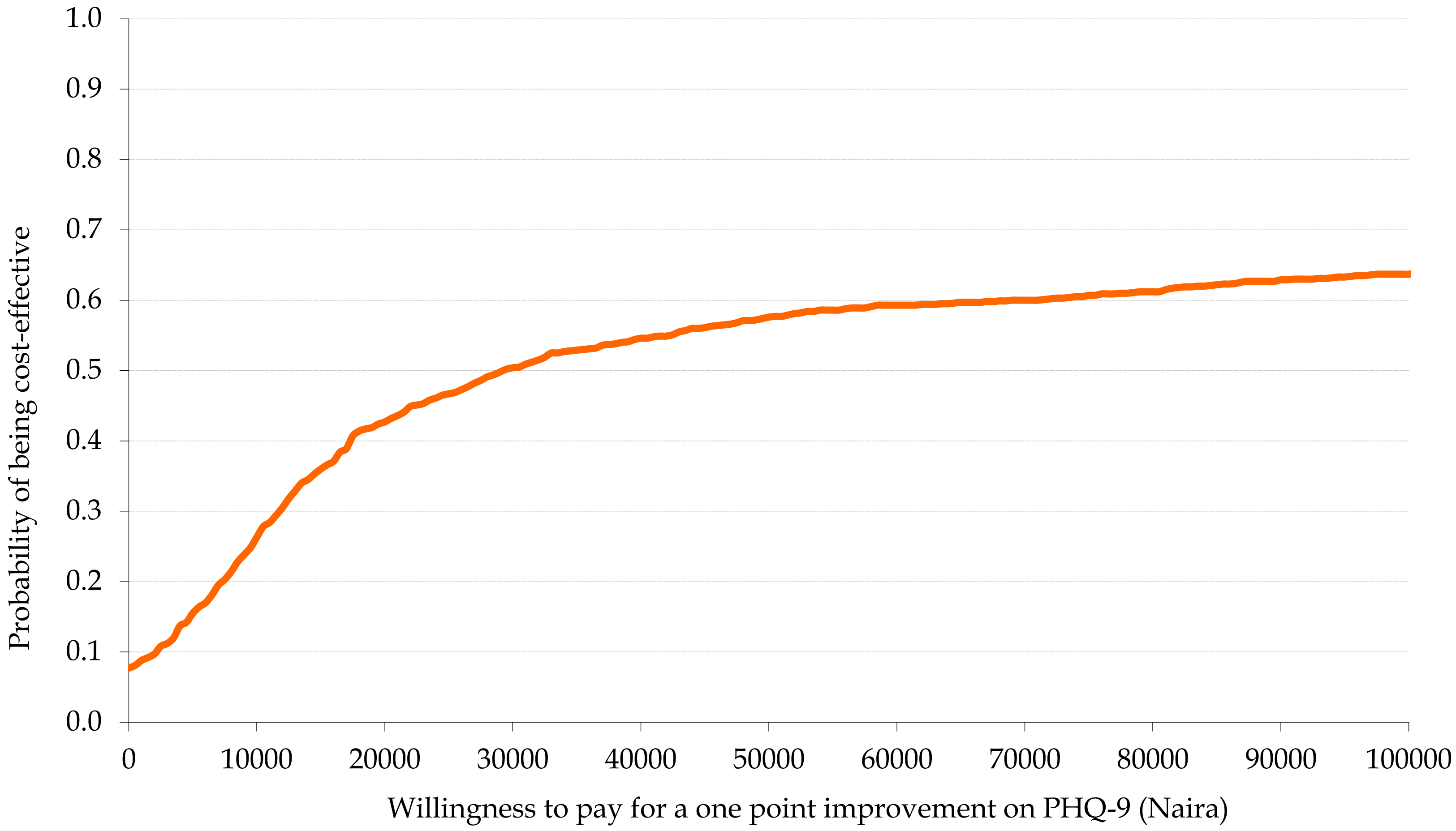

Web Figure 3: Scatterplot of incremental costs (Naira) and effects (WHO-DAS) between STEP versus EUC at 12 months

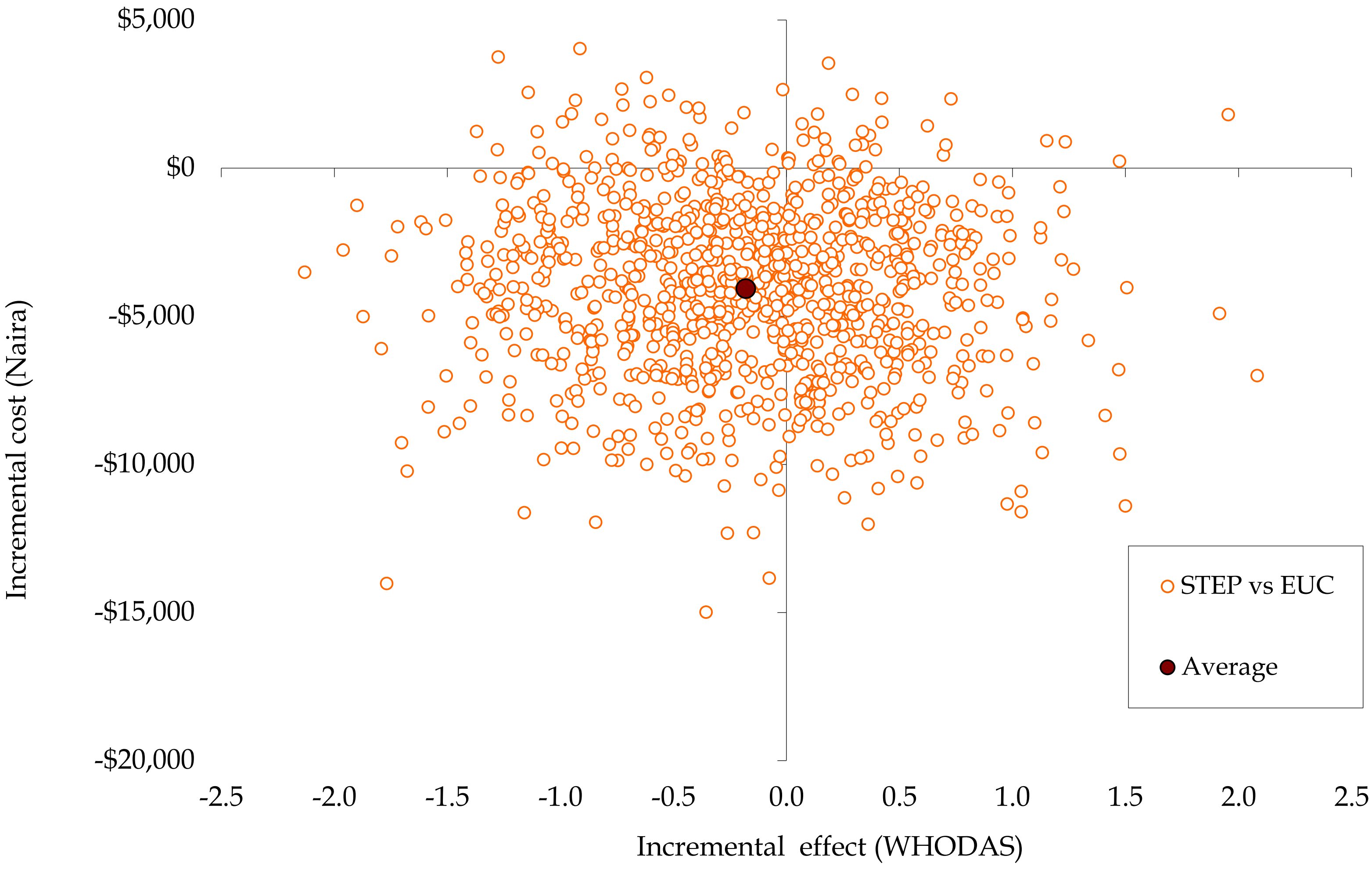

Web Figure 4: Cost-effectiveness acceptability curve showing probability that STEP is a cost-effective intervention compared to EUC at different levels of willingness-to-pay for a one point improvement on WHODAS (in Naira) at 12 months follow-up

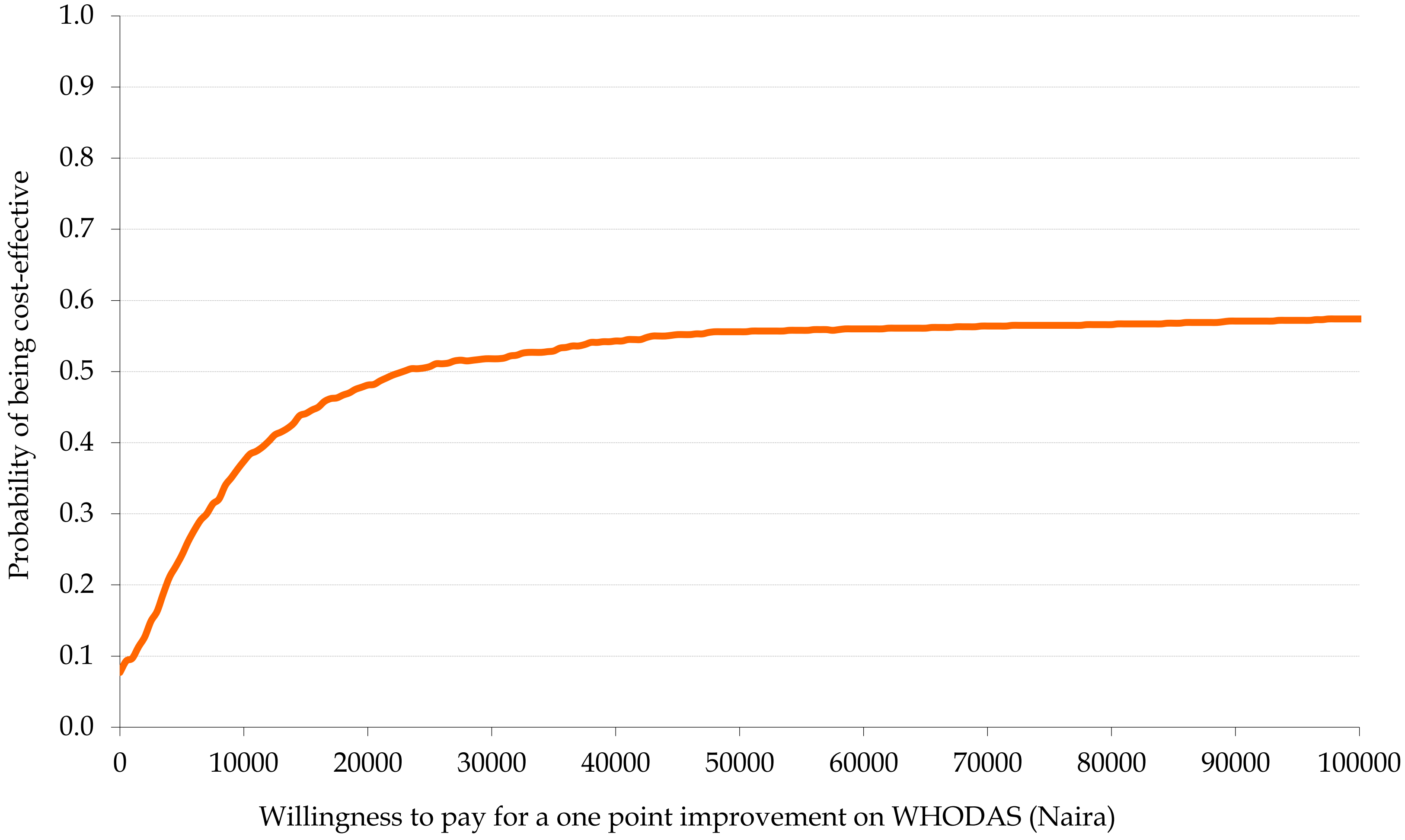

Supplement: Supplementary appendix [file mmc1.pdf]
